# Supplementary material for: Purification, Composition, and Anti-Inflammatory Activity of Polyphenols from Sweet Potato Stems and Leaves
Source: Foods. 2025 Aug 21;14(16):2903. doi: 10.3390/foods14162903 (PMC12385905; doi:10.3390/foods14162903)
Supplement: Supplementary file 1 [file foods-14-02903-s001.zip › foods-3816323-supplementary.pdf]

Table S1. Sequences of primers used for qRT-PCR

Gene	Forward primer	Reverse primer
M-IL-6	CTTCTTGGGACTGATGCTGGT	CACAACTCTTTTCTCATTTCCACG
M-IL-1β	GGGCCTCAAAGGAAAGAATCT	GAGGTGCTGATGTACCAGTTGG
M-IL-10	TACAGCCGGGAAGACAATAACT	AGGAGTCGGTTAGCAGTATGTTG
M-TNF-α	CTACTCCCAGGTTCTCTTCAAGG	CTCCCAGGTATATGGGCTCATAC
M-GAPDH	TGAAGGGTGGAGCCAAAAG	AGTCTTCTGGGTGGCAGTGAT

A

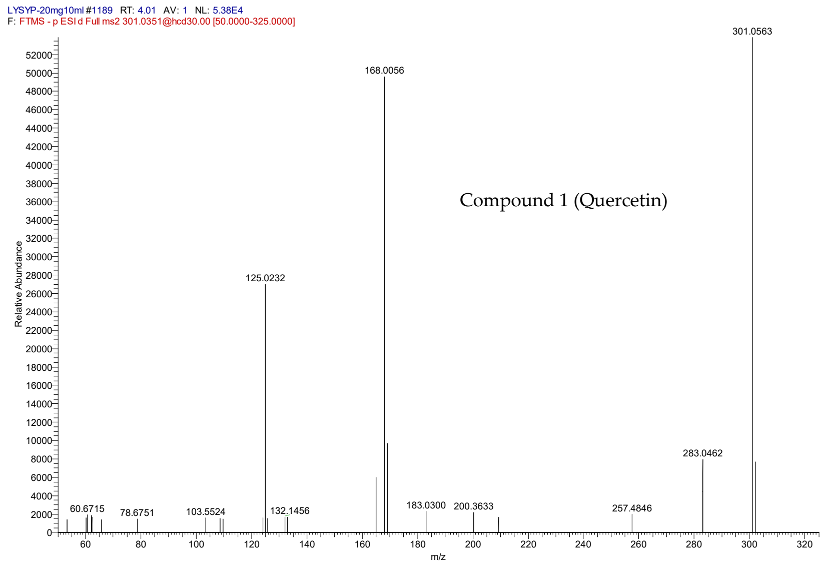

B

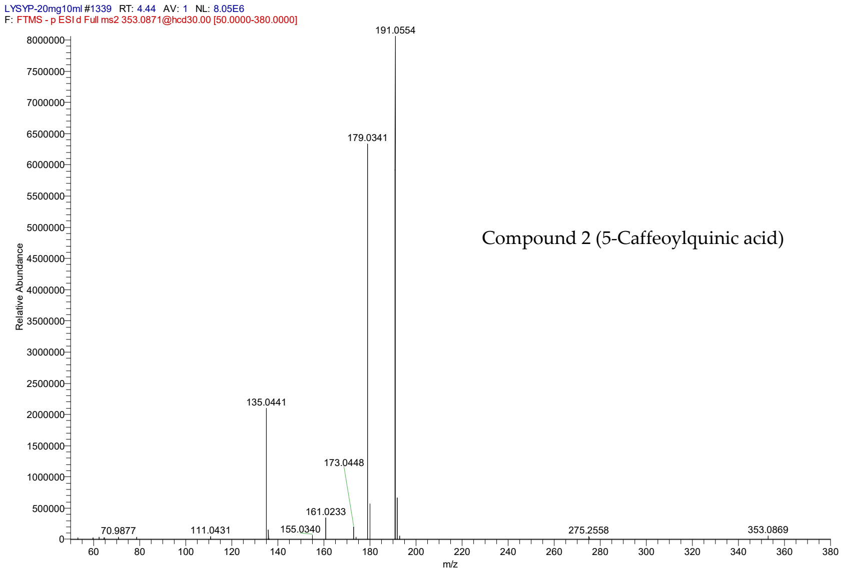

C

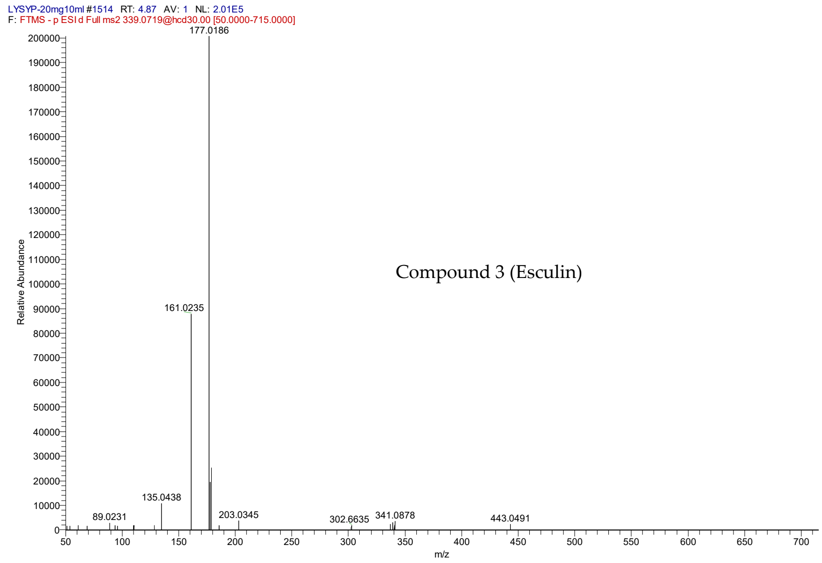
LYSYP-20mg10ml #1514 RT: 4.87 AV: 1 NL: 2.01E5
F: FTMS - p ESI d Full ms2 339.0719@hcd30.00 [50.0000-715.0000]
177.0186
161.0235
135.0438
89.0231
203.0345
302.6635
341.0878
443.0491
Compound 3 (Esculin)
Relative Abundance
m/z

D

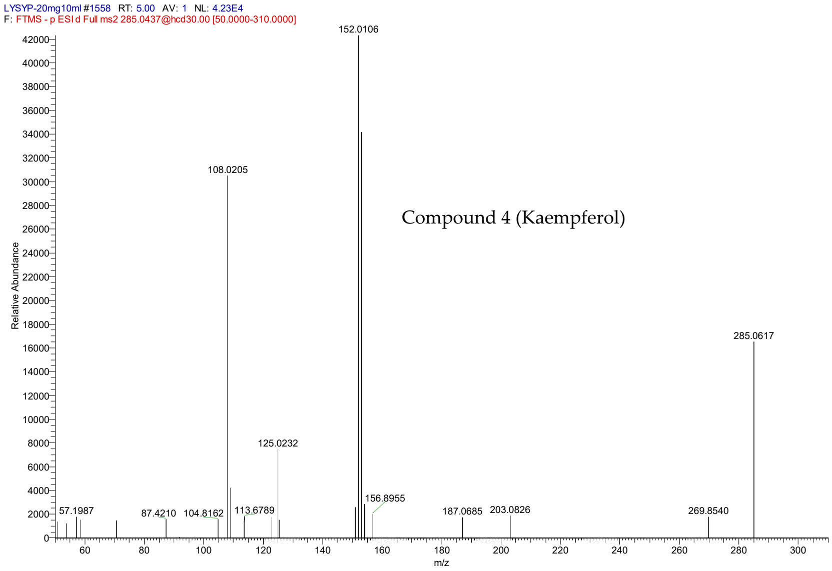
LYSYP-20mg10ml #1558 RT: 5.00 AV: 1 NL: 4.23E4
F: FTMS - p ESI d Full ms2 285.0437@hcd30.00 [50.0000-310.0000]
152.0106
108.0205
Compound 4 (Kaempferol)
285.0617
125.0232
57.1987
87.4210
104.8162
113.6789
156.8955
187.0685
203.0826
269.8540
Relative Abundance
m/z

E

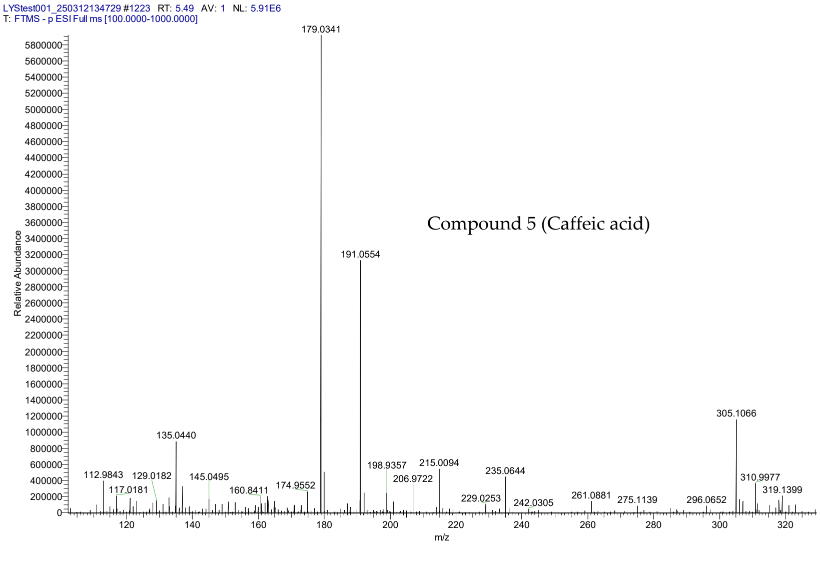
LYStest001_250312134729 #1223 RT: 5.49 AV: 1 NL: 5.91E6
T: FTMS - p ESI Full ms [100.0000-1000.0000]
179.0341
Compound 5 (Caffeic acid)
191.0554
305.1066
135.0440
112.9843
117.0181
129.0182
145.0495
160.8411
174.9552
198.9357
206.9722
215.0094
229.0253
235.0644
242.0305
261.0881
275.1139
296.0652
310.9977
319.1399
Relative Abundance
m/z

F

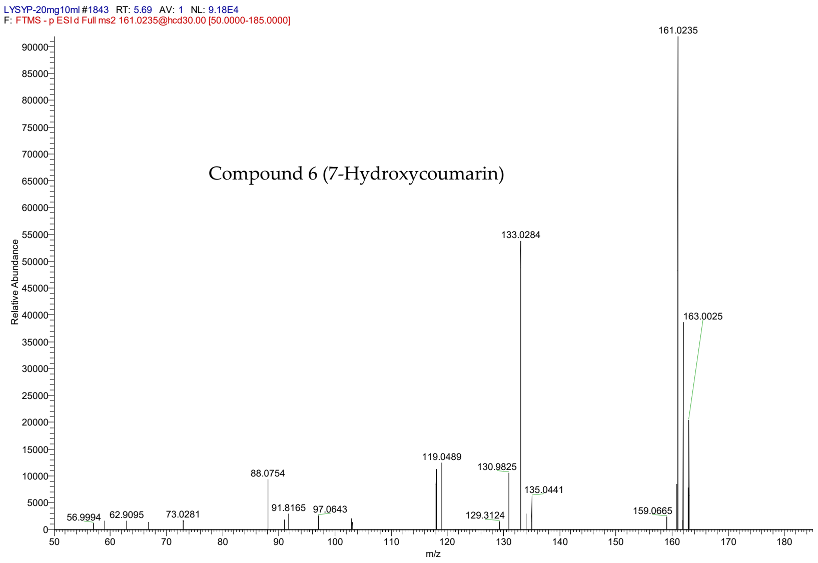
LYSYP-20mg10ml #1843 RT: 5.69 AV: 1 NL: 9.18E4
F: FTMS - p ESI d Full ms2 161.0235@hcd30.00 [50.0000-185.0000]
Compound 6 (7-Hydroxycoumarin)
161.0235
133.0284
163.0025
119.0489
130.9825
135.0441
88.0754
91.8165
97.0643
129.3124
159.0665
56.9994
62.9095
73.0281
Relative Abundance
m/z

G

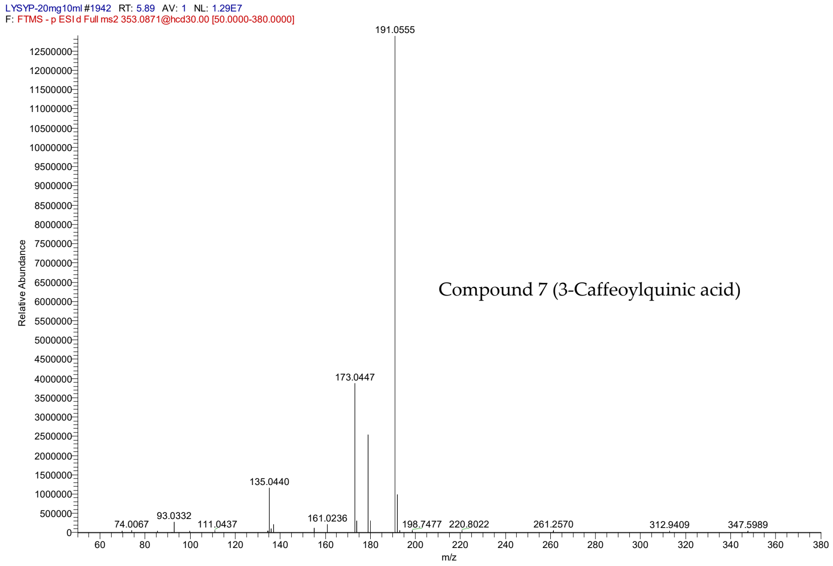
LYSYP-20mg10ml #1942 RT: 5.89 AV: 1 NL: 1.29E7
F: FTMS - p ESI d Full ms2 353.0871@hcd30.00 [50.0000-380.0000]
Compound 7 (3-Caffeoylquinic acid)
191.0555
173.0447
135.0440
74.0067
93.0332
111.0437
161.0236
198.7477
220.8022
261.2570
312.9409
347.5989
Relative Abundance
m/z

H

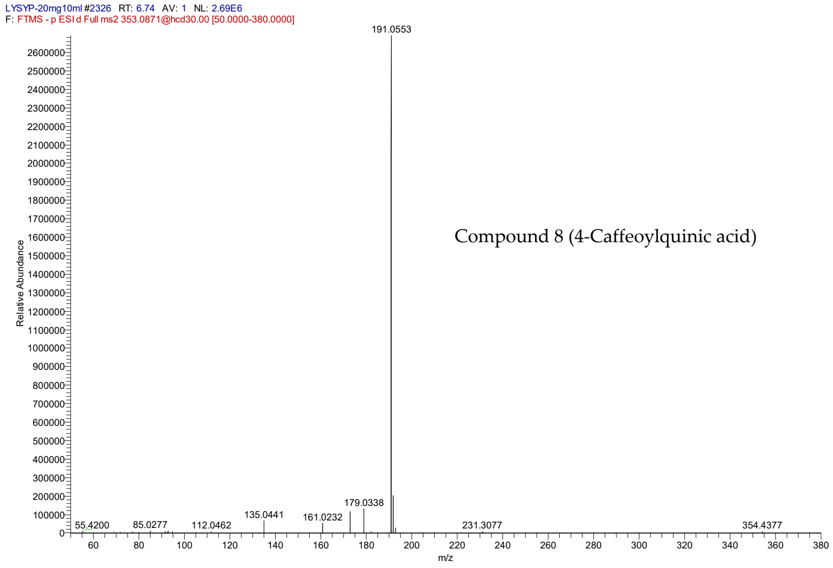
LYSYP-20mg10ml #2326 RT: 6.74 AV: 1 NL: 2.69E6
F: FTMS - p ESI d Full ms2 353.0871@hcd30.00 [50.0000-380.0000]
Compound 8 (4-Caffeoylquinic acid)
191.0553
179.0338
135.0441
161.0232
55.4200
85.0277
112.0462
231.3077
354.4377
Relative Abundance
m/z

I

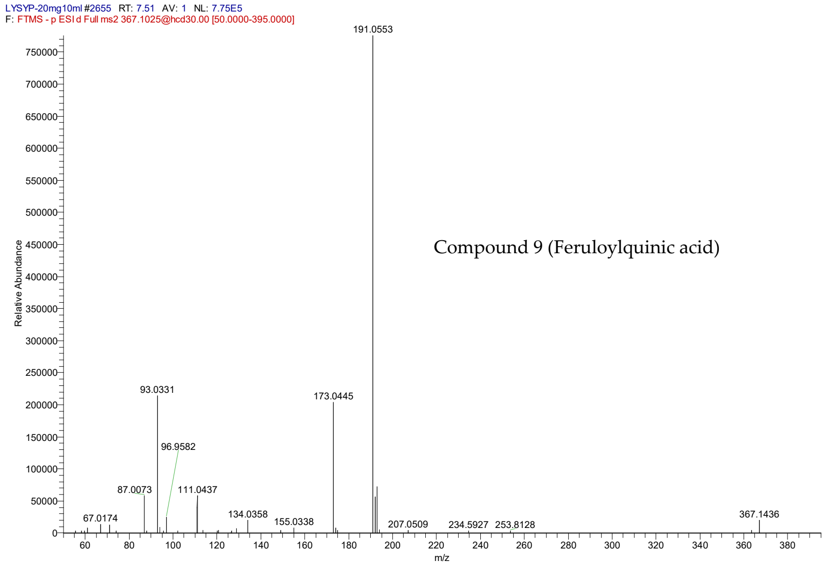
LYSYP-20mg10ml #2655 RT: 7.51 AV: 1 NL: 7.75E5
F: FTMS - p ESI d Full ms2 367.1025@hcd30.00 [50.0000-395.0000]
Relative Abundance
m/z
191.0553
173.0445
93.0331
96.9582
87.0073
111.0437
67.0174
134.0358
155.0338
207.0509
234.5927
253.8128
367.1436
Compound 9 (Feruloylquinic acid)

J

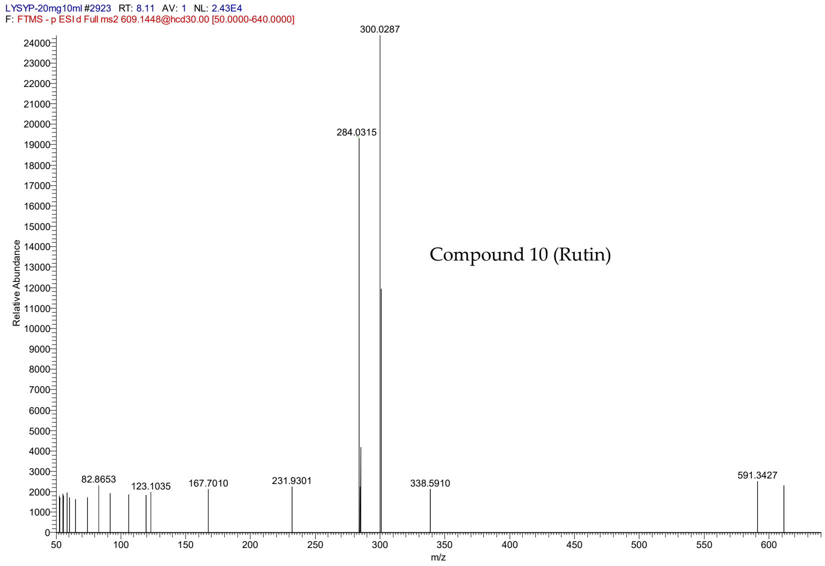
LYSYP-20mg10ml #2923 RT: 8.11 AV: 1 NL: 2.43E4
F: FTMS - p ESI d Full ms2 609.1448@hcd30.00 [50.0000-640.0000]
Relative Abundance
m/z
300.0287
284.0315
82.8653
123.1035
167.7010
231.9301
338.5910
591.3427
Compound 10 (Rutin)

K

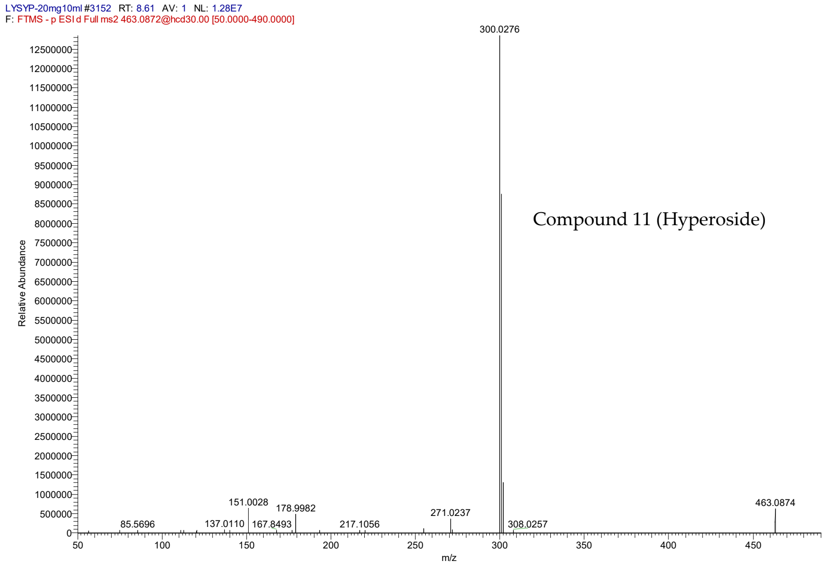
LYSYP-20mg10ml #3152 RT: 8.61 AV: 1 NL: 1.28E7
F: FTMS - p ESI d Full ms2 463.0872@hcd30.00 [50.0000-490.0000]
Relative Abundance
m/z
300.0276
151.0028
178.9982
85.5696
137.0110
167.8493
217.1056
271.0237
308.0257
463.0874
Compound 11 (Hyperoside)

L

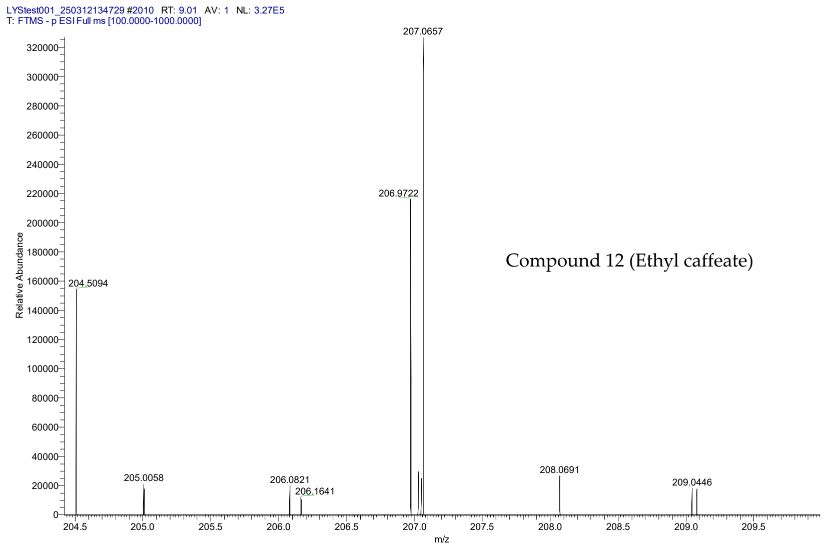
LYStest001_250312134729 #2010 RT: 9.01 AV: 1 NL: 3.27E5
T: FTMS - p ESI Full ms [100.0000-1000.0000]
Relative Abundance
m/z
207.0657
206.9722
204.5094
205.0058
206.0821
206.1641
208.0691
209.0446
Compound 12 (Ethyl caffeate)

M

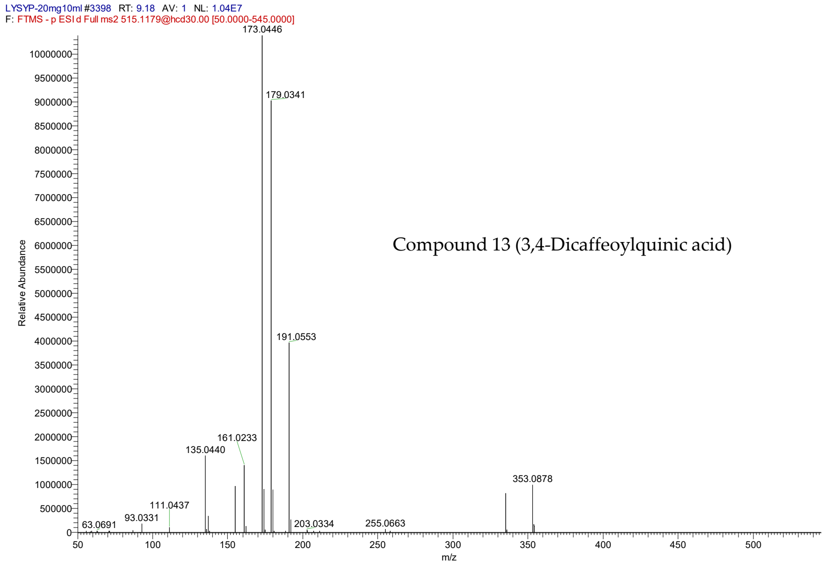
LYSYP-20mg10ml #3398 RT: 9.18 AV: 1 NL: 1.04E7
F: FTMS - p ESI d Full ms2 515.1179@hcd30.00 [50.0000-545.0000]
Relative Abundance
m/z
173.0446
179.0341
191.0553
161.0233
135.0440
111.0437
93.0331
63.0691
203.0334
255.0663
353.0878
Compound 13 (3,4-Dicaffeoylquinic acid)

N

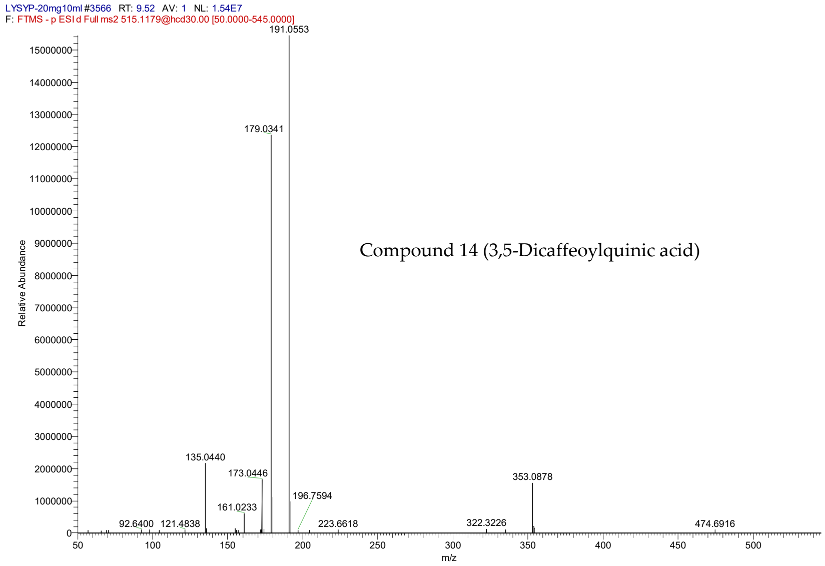
LYSYP-20mg10ml #3566 RT: 9.52 AV: 1 NL: 1.54E7
F: FTMS - p ESI d Full ms2 515.1179@hcd30.00 [50.0000-545.0000]
Relative Abundance
m/z
191.0553
179.0341
135.0440
173.0446
196.7594
161.0233
92.6400
121.4838
223.6618
322.3226
353.0878
474.6916
Compound 14 (3,5-Dicaffeoylquinic acid)

O

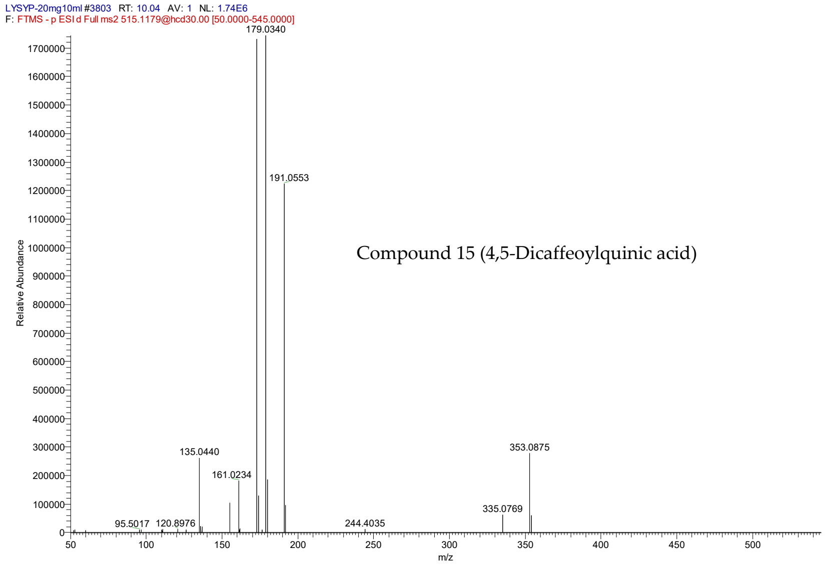
LYSYP-20mg10ml #3803 RT: 10.04 AV: 1 NL: 1.74E6
F: FTMS - p ESI d Full ms2 515.1179@hcd30.00 [50.0000-545.0000]
Compound 15 (4,5-Dicaffeoylquinic acid)
179.0340
191.0553
135.0440
161.0234
353.0875
335.0769
95.5017
120.8976
244.4035
Relative Abundance
m/z

P

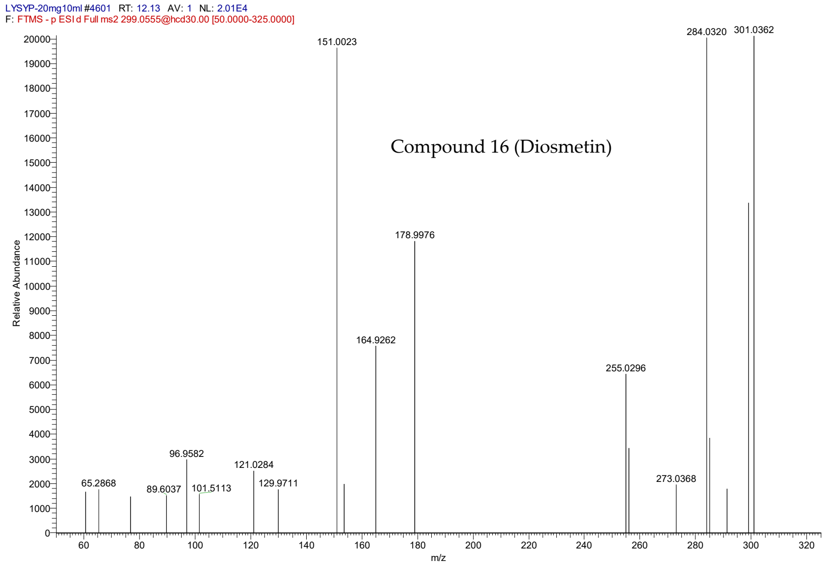
LYSYP-20mg10ml #4601 RT: 12.13 AV: 1 NL: 2.01E4
F: FTMS - p ESI d Full ms2 299.0555@hcd30.00 [50.0000-325.0000]
Compound 16 (Diosmetin)
151.0023
284.0320
301.0362
178.9976
164.9262
255.0296
96.9582
121.0284
65.2868
89.6037
101.5113
129.9711
273.0368
Relative Abundance
m/z

Q

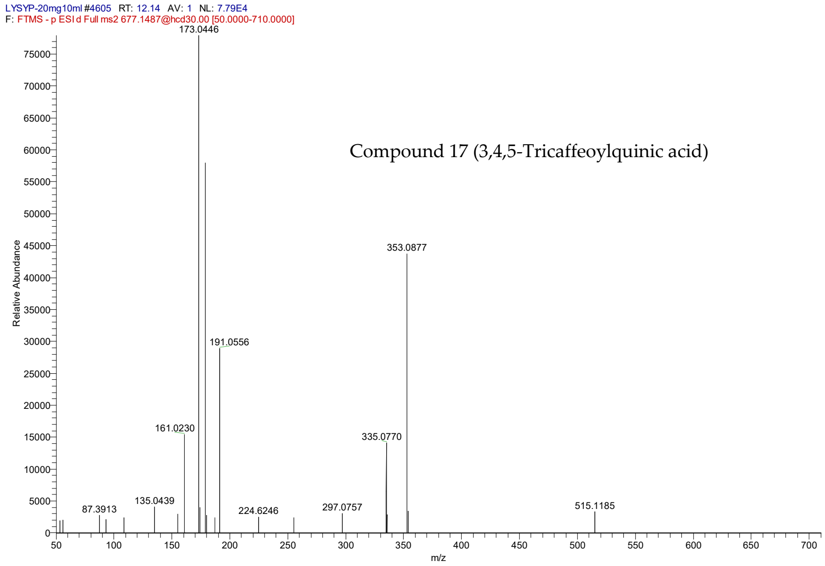
LYSYP-20mg10ml #4605 RT: 12.14 AV: 1 NL: 7.79E4
F: FTMS - p ESI d Full ms2 677.1487@hcd30.00 [50.0000-710.0000]
Compound 17 (3,4,5-Tricaffeoylquinic acid)
173.0446
353.0877
191.0556
161.0230
335.0770
87.3913
135.0439
224.6246
297.0757
515.1185
Relative Abundance
m/z

R

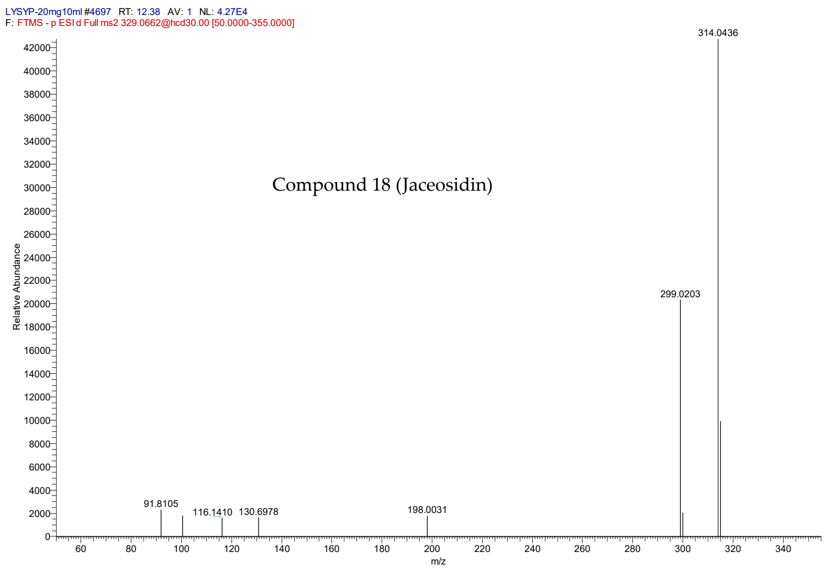

S

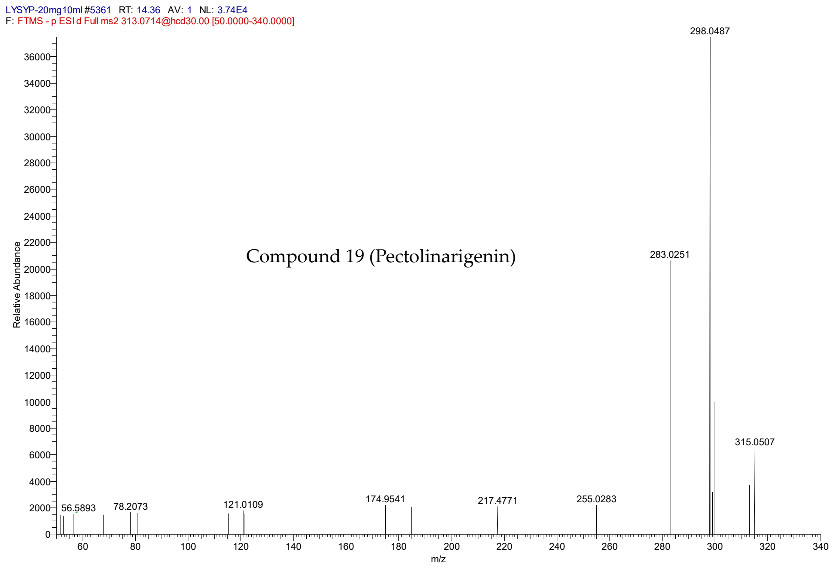

Figure S1. The precursor and fragment ion mass spectra of 12 phenolic acids and 7 flavonoids. A: Quercetin; B: 5-Caffeoylquinic acid; C: Esculin; D: Kaempferol; E: Caffeic acid; F: 7-Hydroxycoumarin; G: 3-Caffeoylquinic acid; H: 4-Caffeoylquinic acid; I: Feruloylquinic acid; J: Rutin; K: Hyperoside; L: Ethyl caffeate; M: 3,4-Dicaffeoylquinic acid; N: 3,5-Dicaffeoylquinic acid; O: 4,5-Dicaffeoylquinic acid; P: Diosmetin; Q: 3,4,5-Tricaffeoylquinic acid; R: Jaceosidin; S: Pectolinarigenin.

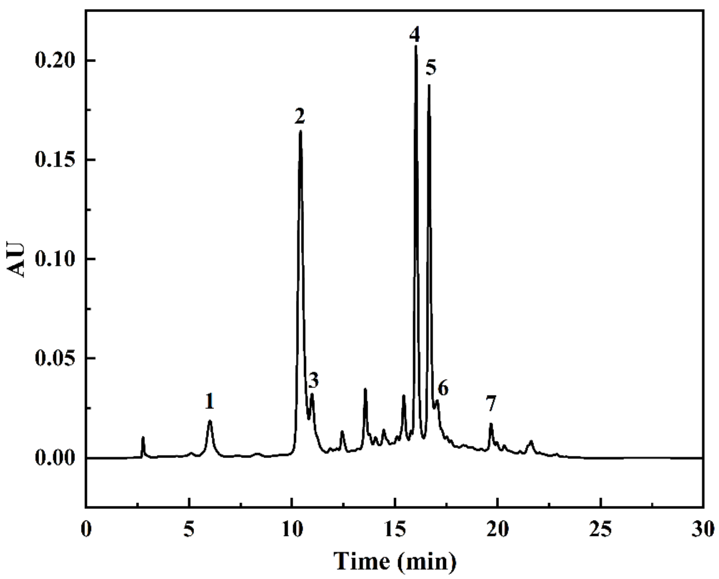

Figure S2. HPLC chromatogram of chlorogenic acids in purified SPSL polyphenols. Peak 1: 5-CQA, peak 2: 3-CQA, peak 3: 4-CQA, peak 4: 3,4-diCQA, peak 5: 3,5-diCQA, peak 6: 4,5-diCQA, peak 7: 3,4,5-triCQA.
